# Reducing Sodium Content in Cheeses While Increasing Salty Taste and Fat Perception Using Aroma

**DOI:** 10.3389/fnut.2022.873427

**Published:** 2022-07-08

**Authors:** Adiansyah Syarifuddin, Chantal Septier, Christian Salles, Thierry Thomas-Danguin

**Affiliations:** CSGA (Centre des Sciences du Goût et de l’Alimentation), CNRS, INRAE, Institut Agro, Université de Bourgogne Franche-Comté, Dijon, France

**Keywords:** odor, cheese, fat, salt, cross-modal perceptual interaction, perception

## Abstract

Excess salt (NaCl) and fat intake are major causes of chronic diseases, but reducing such components without affecting acceptability is a major challenge. Here, we set out to examine whether added aroma in lower salt cheese can enhance saltiness and fat perception. Low-salt cheese samples were grated through a homogenizer, and then aroma solution, sardine aroma (salt-associated), butter aroma (fat-associated) and a mix of sardine and butter aromas were added. The results confirmed that grating changes cheese texture, leading to induced taste perception. In addition, a significant saltiness enhancement was induced by sardine aroma and to a lesser extent by butter aroma, while significant fat perception enhancement was only induced by blended aroma. These findings show that aroma addition can be a strategy to compensate for sodium reduction in commercial cheese. Concerning fat perception, the addition of aroma can be a good strategy to compensate for low-fat in commercial cheeses. However, the mechanisms involved seem complex and need to be elucidated.

## Introduction

In the development of food products, a reduced amount of salt has been proposed by international and national authorities because higher salt intake has been linked to the development of many diseases such as hypertension, cardiovascular disease and other health problems (1–5). For example, in the United States, the current average intake of sodium is 3,400 mg/d, whereas 2,300 mg/d is the current recommendation for healthy adults aged < 50 years, and 1,500 mg/d is recommended for those at risk of cardiovascular disease and/or aged ≥ 50 years (6). For optimal heart health, the American Heart Association recommends that people eat no more than 1,500 mg of sodium per day (7).

Components of cheese, such as salt, fat, protein, and water, are known to play important roles in cheese production (8). Salt is traditionally added in varying amounts depending on the cheese variety (9). It is an important operation in the cheese making process because it adds the final organoleptic properties such as the flavor and texture as well as color of cheese, prevents microbial growth and acid development and adjusts the moisture content of the cheese by forcing moisture out (10).

The acceptability of food is mainly related to its texture, flavor, and appearance, which are also determinant factors for consumer preference and demand for food (11). Cheeses contain complex mixtures of hundreds of volatile compounds, including short-chain carboxylic acids, sulfur compounds, esters, alcohols, ketones, and lactones (12–14), which contribute to the overall sensory perception of flavor-related attributes.

Thus, any treatment involving further reduction of salt in ready meals is requested by the food industry to be in line with the recommendations. However, neither consumer nor food manufacturers are willing to accept strategies that sacrifice the sensory and nutritional value of products.

Various methods have been applied by food manufacturers as they attempt to reduce the amount of salt in their products. The first method is to gradually apply changes to food ingredients without consumer noticing (15). This strategy has been successfully applied for salt reduction in bread (16). However, the gradual reduction of salt content in food has been time-consuming, and the loss of overall flavor will be recognized by consumers after the degree of salt reduction is reached (17). Potassium chloride is the most commonly used salt substituent to reduce sodium levels in different foods. However, potassium chloride has been shown to impart bitter and/or metallic taste to foods when used at high levels, thus limiting its replacement (17, 18). In the case of cheeses, the use of salt substitutes to replace sodium cations with potassium, calcium or magnesium often results in changes in texture and taste perception (19, 20). Several strategies have been proposed to reformulate foods with a lower sodium content (21, 22). However, it remains a challenge.

Another possible approach to reduce sodium contents in processed foods is to use tasteless aroma compounds. This original approach is based on multisensory integration mechanisms (23). Integration across sensory modalities is reflected in the presence of multimodal neurons that receive converging sensory information (24, 25). Many studies have shown the impact of olfactory stimuli on taste perception. It has been reported that strawberry odor can enhance sweetness perception and soy sauce odor can enhance saltiness perception by both perceived and imagined odors (26). Several studies have reported the ability of food odors to increase taste perception (27). The taste component could be evoked when an odor component of a familiar food was experienced (28). Moreover, cross-modal taste-smell interactions depended not only on odor-taste congruency but also on taste compound concentration, as reported for saltiness perception enhancement (29). Panelists can estimate food saltiness on the basis of their written names (30). Moreover, saltiness perception can be enhanced using salt-associated odors in simple water solutions (30) and in model cheeses (31). More recently, using model cheeses varying in composition and texture, it was reported that salt-associated aroma (sardine) and fat-associated aroma (butter) were able to enhance saltiness perception and fat perception, respectively, but these effects were highly dependent on the composition and structure of the products, for which the role appeared to be complex (32). Moreover, these authors reported that the butter aroma also modulated the saltiness perception. To date, very few studies have reported the contribution of well-selected aromas to enhance taste perception of complex foods such as saltiness and fat perception for dairy products. However, this phenomenon can be positively used as a strategy to compensate for the taste perception of low-salt cheeses. Therefore, the aim of this study is to examine whether salt content can be reduced in cheeses and whether aromas can help to maintain saltiness and fat perception for consumers in low-salt cheeses. To do so, we followed a sensory approach using flavored semihard cheese and involving untrained panelists.

## Materials and Methods

### Materials

The commercial cheeses used in this study were a regular salt cheese (RSC) and a low-salt cheese (LSC, –33% salt) from Orval Abbey (Villers-devant-Orval, Belgium). These are semihard cheeses, pertaining to the “Trappist” cheese category. For RSC as for LSC, dry matter was 54%, fat in dry matter: 58%, moisture on fat-free cheese: 61%, fat content: 32%, pH: 5.24. Salt content was 1.78% for RSC and 1.19% for LSC. RSC and LSC were made with pasteurized cow milk, rennet, lactic ferment, and salts. Salts were incorporated in the cheese curd by brining during different times. LSC and RSC came each one from the same brining batch. In addition, after grating, the pieces of cheese were mixed to obtain portions whose homogeneity in salt content was controlled.

A salt-congruent complex aroma, reminiscent of the odor of smoked sardine (Givaudan, Argenteuil, France) and further called the “sardine aroma,” and a fat-congruent aroma compound, 2,3-pentanedione (Sigma–Aldrich, Saint Quentin Fallavier, France), reminiscent of the odor of butter and further called the “butter aroma,” were used to flavor the low-salt cheeses. These aromas were dissolved in propylene glycol (Sigma–Aldrich). All materials were food-grade quality.

### Preparation of Flavored and Unflavored Cheese Samples

Modified versions of the commercial cheeses were developed in the laboratory. We specifically designed low-salt flavored cheese by adding 0.5% propylene glycol (PG), used as a solvent of aroma, in which was dissolved 5% sardine aroma, 1% butter aroma or a blend of these two aromas (1.3% sardine aroma and 0.8% butter aroma). The final aroma concentrations were chosen in order to have close aroma intensities and were of 250 ppm for sardine aroma in low-salt cheese (LSC Sardine), 50 ppm for butter aroma in low-salt cheese (LSC Butter), and 65 ppm for sardine aroma and 40 ppm for butter aroma in low-salt cheese (LSC Blended).

To design the flavored cheeses, commercial cheeses were first grated by passing the samples through a homogenizer (R3V.V, Robot-coupe, Montceau en Bourgogne, France) for 30 s to grate the cheeses at room temperature. The aroma was added to the grated cheese, which was mixed again several times for 20 s, with 10 s rest intervals until the mixture was homogenous. The product mixture was then placed into a vacuum plastic bag, rolled, and vacuum sealed with a vacuum machine (System CT 100, SASA Bodson Industrie, Le Cateau Cambrésis, France) until a pressure of –0.8 bar, identical for all samples, and finally stored at 4°C for 2 days to allow the cheese to become compact again. The aroma concentrations used in this study were chosen according to their intensity and acceptability in a preliminary study involving an internal panel (data not shown).

As a control, unflavored products were also prepared. These samples were called LSC-grated and RSC-grated and followed exactly the same process except for the addition of aroma. These control samples help to understand the impact of grating on salt release. In addition, because PG has been used as the solvent for aroma, two other control samples were prepared. They were called LSC-PG and RSC-PG and followed the same process as flavored cheeses, but instead of adding aroma, only 0.5% PG was added.

All grated cheese samples were stored at 4°C for 2 days before testing. For safety reasons, the tools used during cheese preparation were disinfected with 70% ethanol. Each sample was checked for the absence of total coliforms, *L. monocytogenes*, *Salmonella*, and *Staphylococcus* for each batch by an accredited laboratory (Laboratoire Départemental de Côte d’Or, Dijon, France).

### Sensory Analysis

#### Panelists

Fifty-seven panelists (aged 20–61 years, 42 women, 15 men) of the Dijon population were recruited for this experiment. All participants were able to recognize bitter, salty, sour, and sweet tastes in water solutions (L-leucine 8 g/L; sodium chloride 2 g/L; lactic acid 2 mL/L; lactose 35 g/L) and to correctly describe the two target aromas when smelling flavored water solutions (sardine 0.5 g/L; butter 0.02 g/L). Before performing the sensory test, panelists were requested not to smoke or to eat or to drink anything except water at least 1 h before the sensory session. This study was performed in accordance with the relevant institutional and national regulations and legislation (Comité de Protection des Personnes Est-1, N° 2013/64—IDRCB 2013—A01084-41 on 11/21/2013 and Agence Nationale de la Santé et du Médicament, N° B 131283-81 on 11/20/2013). The participants were requested to sign an informed consent form, but they were not informed of the aims of the experiment. They received €70 indemnity for the whole experiment.

#### Measurement Sessions

Each panelist took part in two 1-h sessions. In the first session, they received instructions and performed taste recognition tests and odor description tests. In the test session, each panel had to evaluate 9 samples: RSC, LSC, RSC-grated, LSC-grated, RSC-PG, LSC-PG, LSC-sardine, LSC-butter, and LSC-blended. For each sample, panelists were asked to rate odor and taste intensity (bitterness, saltiness, sourness, sweetness, and aroma intensity) first and then to rate texture attributes (elasticity, moistness, firmness, melting, graininess, and perceived fat content). To perform the rating tasks, panelists used linear scales from 0 to 10 (0: none and 10: extremely strong). An interval of 30 s was imposed between each sample. During this time, panelists were asked to cleanse their mouth with apple and bread and finally rinse the mouth with mineral water (Evian^®^, Danone, France).

Cheese samples were prepared 2 days before each sensory session. Two pieces of 3.5 ± 0.4 g of samples were put in a hermetically closed cup coded with a three-digit code. The samples were served monadically to the panelists in a balanced order across subjects. The tests were conducted in an air-conditioned room (21°C) dedicated to sensory evaluation. The participants were placed in separate booths under red light. Sensory data acquisition was performed with the FIZZ software (Biosystemes, Couternon, France).

### Instrumental Rheological Measurements

Uniaxial compression tests were performed using a Texture Analyzer (TA-XT plus, Stable Microsystems Ltd., Godalming, United Kingdom) equipped with a cylindrical stainless-steel probe (100 mm in diameter) compression platen attachment. Rectangular pieces of cheese (3 cm high, 2 cm wide, 1 cm deep) were sampled and stored at 15°C for 15 min before tests. Tests were performed at 15°C at a constant displacement rate with a crosshead speed of 0.8 mm/min to a maximum deformation of 80%. Five replicates per sample were performed. The force developed by the cheese sample, equal to the resistance of the sample during compression, was measured with a load cell and recorded according to the position of the top plate. Using the force and displacement data recorded, the engineering stress (σ = F_t_/A_0_, F_t_ = recorded force and A_0_ = initial cross section) and Cauchy strain (ε = Δh/h_0_, Δh = displacement and h_0_ = initial height) were calculated (33). From these data, the modules of deformability MD (kPa), fracture stress σf (kPa) and strain ε (dimensionless) and work to fracture Wf (kJ.m^–3^) were calculated.

### Data Analyses

Data analyses were performed with the R software (release 4.0.4). Odor-induced saltiness enhancement (OISE) and odor-induced fat perception enhancement (OIFE) were calculated to evaluate the specific influence of aroma addition on salty taste and fat content perception. For a given sample, the OISE was the mean value of the paired difference (for each panelist) between the saltiness of the flavored sample and the saltiness of the corresponding unflavored sample; then, the grand mean across subjects was calculated. Similarly, the OIFE was the mean value of the paired difference between the fat perception of the flavored sample and the fat perception of the corresponding unflavored sample.

For rheological data, analysis of variance (ANOVA) and multivariate analysis of variance (MANOVA) were performed using the *manova* function (*stats* package). For sensory data, MANOVA was performed with the *manova* function (*stats* package), and approximate F values were reported. ANOVA was performed using a mixed effects model (*nlme* package) with panelists as a random factor. *Post hoc* tests were performed through a multiple comparison of means with the Bonferonni correction. For all data analyses, the effects were considered to be significant when *p* < 0.05.

## Results

### Impact of Aroma Addition on Rheological Properties

Rheological measurements were performed on low-salt cheese samples to evaluate the impact of aroma addition on product structure (LSC sardine, LSC butter, LSC blended; LSC PG as a control) (data not shown). One-way ANOVA, with products as a factor, was performed on each rheological parameter, and the results showed that aroma addition had no significant effect (*p* > 0.05) on the product structure.

### Impact of Grating on Texture and Taste Perception

**FIGURE 1:**
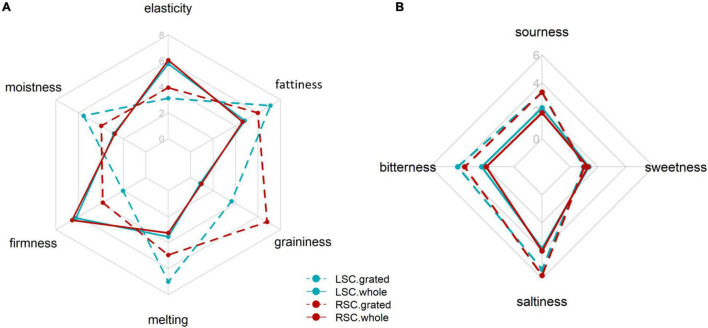
Texture **(A)** and taste **(B)** profile of *whole* cheeses and *grated* cheeses at a regular (RSC) and low (LSC) salt content.

For the flavoring process of the cheese, a grating step was required. Therefore, we first evaluate the effect of grating on perception. Four products (LSC-whole, RSC-whole, LSC-grated and RSC-grated) were considered as a function of salt level (regular or low) and grating status (whole or grated). A two-way MANOVA (salt level and grating status as factors) was performed on texture perception attributes (elasticity, moistness, firmness, melting, graininess, perception of fat content). The effects of grating on texture and taste attributes are reported in Figures 1A,B, respectively. The obtained results indicated a significant effect of salt level [*F*(6, 219) = 11.1, *p* < 0.0001] and grating [*F*(6, 219) = 82.3, *p* < 0.0001]. The interaction between these factors was also significant [*F*(6, 219) = 8.6, *p* < 0.0001]. In addition, the impact of grating on taste perception (sour, bitter, salty, sweet) was also investigated. The MANOVA results showed that salt level was not significant [*F*(4, 221) = 0.82, *p* = 0.5], but grating was significant [*F*(4, 221) = 12.6, *p* < 0.0001]. The interaction between factors was not significant [*F*(4, 221) = 0.25, *p* = 0.9].

Six separate two-way ANOVAs (salt level and grating as fixed factors; panelists as random factors) were performed on each texture and taste attribute (Figure 1 and Table 1). Grating had a significant influence on all sensory attributes of texture and taste. Salt level had a significant influence on all texture attributes, most of the time in interaction with the grating condition. However, Figure 1A shows that the interaction is mostly due to large differences between the low and regular salt levels for the grated cheese. Surprisingly, salt level had no significant effect on taste attributes, even if low-salt cheeses tended to be less salty and more bitter.

**TABLE 1:** Sensory properties of regular and low salt semihard cheeses: Fisher statistics from ANOVA on the sensory attribute intensity (unflavored cheeses).

	Salt level		Grating condition		Salt*Grating	
	*F*(1, 168)	*p*	*F*(1, 168)	*p*	*F*(1, 168)	*p*
Elasticity	3.91	0.049	74.6	<10^–4^		ns
Moistness	16.0	10^–4^	91.3	<10^–4^	13.1	0.0004
Firmness	24.3	<10^–4^	255	<10^–4^	11.4	0.0009
Melting	22.4	<10^–4^	108	<10^–4^	12.8	0.0005
Graininess	54.0	<10^–4^	379	<10^–4^	47.6	<10^–4^
Fattiness	10.8	0.0012	85.4	<10^–4^	5.46	0.0206
Saltiness		ns	44.8	<10^–4^		ns
Sourness		ns	35.6	<10^–4^		ns
Bitterness		ns	44.9	<10^–4^		ns
Sweetness		ns	5.76	0.017		ns

*ns, not significant.*

To evaluate whether propylene glycol (flavor solvent) could influence the taste characteristics of the cheeses (LSC-PG, LSC-grated), a one-way MANOVA on taste descriptors (sour, bitter, salty, sweet) was performed. The results showed that the addition of PG did not significantly influence the perception of taste (*p* > 0.05) (Results not shown).

### Taste-Aroma Interaction

**FIGURE 2:**
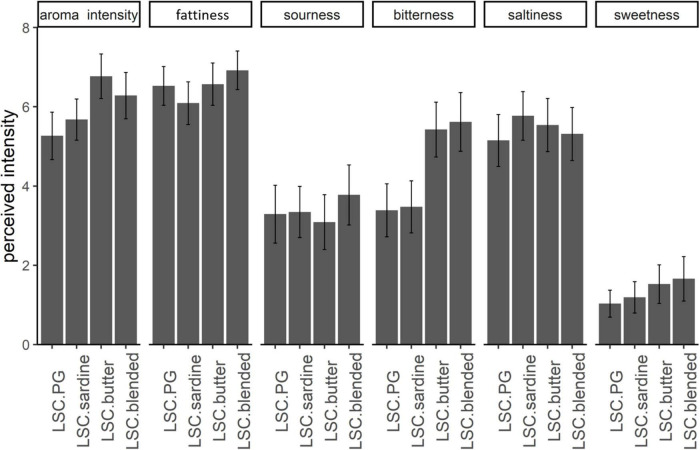
Mean perceived aroma and tastes intensity for aroma-added (sardine, butter or blended) low-salt cheeses and no aroma-added (LSC. PG) cheese. Error bars indicate 95% confidence intervals on the mean.

To assess the influence of odor perception on taste perception, 4 products (LSC-sardine, LSC-butter, LSC-blended and LSC-PG as a control) were considered. A two-way MANOVA was performed on taste descriptors with the type of aroma and aroma intensity as factors. The obtained results showed that the factor type of aroma was significant [*F*(15, 654) = 3.0, *p* = 0.0001; Figure 2]. Aroma intensity also has a significant influence on taste [*F*(5, 216) = 10.9, *p* < 0.0001]. The interaction between the two factors was not significant [*F*(15, 654) = 0.78, *p* = 0.7).

**FIGURE 3:**
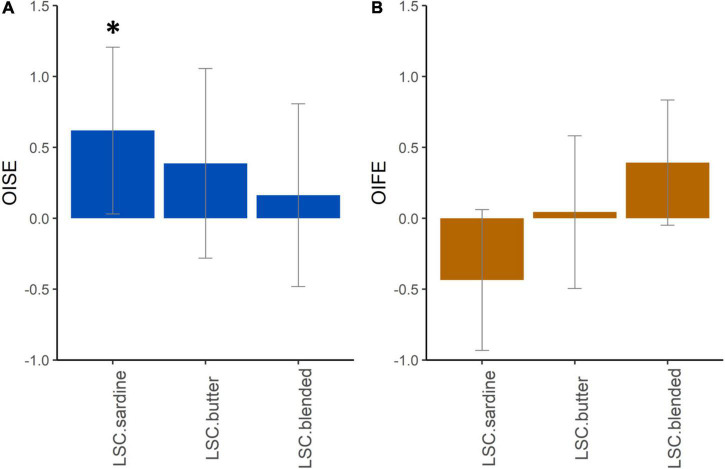
Mean odor-induced saltiness enhancement (OISE; **A**) and odor-induced fat perception enhancement (OIFE; **B**) for low-salt cheeses flavored with a sardine, butter or blended aroma. Error bars indicate 95% confidence intervals on the mean. The star indicates a statistically significant OISE (*p < 0.05).

To evaluate the increase in saltiness and perceived fat induced by odor perception in this study, odor-induced saltiness enhancement (OISE) and odor-induced fat perception enhancement (OIFE) were calculated for LSC sardine, LSC butter, and LSC blended. The significance of the obtained results on both OISE and OIFE was evaluated through 95% confidence intervals of grand means (Figures 3A,B). The obtained results showed that sardine aroma significantly enhanced saltiness perception (*p* < 0.05), whereas it tended to decrease fat perception. OIFE was the highest for blended aroma, whereas butter aroma had no significant effect on OIFE.

## Discussion

The aim of this study was to investigate whether added aroma could help to enhance salt and fat perception in a low salt commercial cheese. Very few studies on cross-modal perceptual interactions have been performed on food products, but most of them have been performed on simple liquid or solid models. In the case of cheese, the main technical difficulty was to incorporate extrinsic odorants into the cheese matrix that was as homogeneous as possible. Because it was impossible to add aroma in the initial steps of cheese making and to keep added aroma compounds safe during the entire maturation process, we chose to grate the cheese in small particles before adding a defined quantity of aroma compounds. However, this process modified taste and texture perception since breakdown of the main constituents of cheese matrix curd is responsible for changes in texture, taste, and aroma. Clearly, the rheological and textural properties of the flavored cheese products were different from those of the original products, although they were recompressed under vacuum. Nevertheless, these properties remained comparable between all grated products concerning sensory properties, which was important for this research work.

We observed that grating significantly increased the saltiness intensity of the grated cheeses compared to the whole cheeses (Figure 1). This previously reported phenomenon (34, 35) can be explained by an increase in mineral salt availability in grated cheeses. In general, an increasing cheese surface area leads to a higher release of flavor compounds. It was reported a clear increase in saltiness perception in relation to the cheese structure deconstruction level but also a decrease in bitterness, while sourness remained unchanged (34). In contrast, an increase in bitterness and sourness was observed with grating in this study. This result can be explained by the difference in the nature of the food matrix but also by higher concentrations of bitter and acid compounds, which are also more easily released in saliva because of the higher exchange surface. Concerning texture perception, as expected, the effect of grating led to a higher graininess and lower elasticity and firmness perception. The higher perceived moistness could be interpreted as a higher and quicker hydration capacity of the fine particles of cheese compared to the whole cheese, which should be progressively broken down and hydrated by saliva. The grated cheeses were also perceived to be more fatty and melty. During grating, the structure of cheeses was crushed by shearing, which could lead to changes in the physical state and distribution of fat, in particular decreasing fat globule size and protein and water distribution (36–38). Moreover, this could lead to a higher availability of fat globules in the mouth.

Our results showed that the taste dimension was not affected by salt level in unflavored cheese, which suggests that low-salt cheeses (–30% salt content) have been perceived to be as salty as regular-salt products (Table 1 and Figure 1). In other words, the 30% salt reduction in the low-salt product has gone unnoticed by the panelists. A similar observation was reported in a study on cheddar cheese (39). The authors did not observe any flavor or liking difference between products, but only a slight decrease of saltiness intensity between a typically marketed Cheddar containing 1.44% salt and a reduced salt cheddar containing 1.12% salt. However, at lower salt concentrations, they reported a drastic decrease in saltiness intensity with an increase in bitterness and unpleasant after-tastes. A hypothesis to explain these results can be that a saturation of the salt-targeted receptors occurred at high salt concentrations in the mouth so that for the high salt level cheeses, a reduction of 30% remained unnoticed. Another hypothesis could be an increase of saltiness perception due to taste-taste interactions (40) because bitterness is slightly enhanced in LSC. More recently, (41) showed similar results with a low impact on saltiness perception for salt reduction at high salt concentrations but with a more significant effect at lower salt concentrations with increasing off-flavors. In addition, (42) reported that it is possible to reduce salt concentration by 20% in a complex model cheese flavor mixture without significantly affecting the overall cheese flavor intensity, although salt has a larger effect on cheese flavor intensity, thus showing that other flavor components of cheese, such as aroma, could compensate for salt reduction.

Few studies reported perceptual interactions between fat and salt perceptions. In flavored model cheeses varying in fat and salt contents, it was reported that the products containing salt were perceived fattier than the products without added salt, which led to conclusion on the existence of both physicochemical and cross-modal perceptual interactions between fat and salt (43). In a more specific study on salt and fat reduction in model cheeses, the salt content was found to highly influence the perception of fat content: the more salt, the more fat was perceived. But, salt displayed only a small effect on rheological parameters, suggesting that the effect of the salt content on fat perception may result from the perceptual and/or cognitive interactions in the salty food (32). Interestingly, it was also reported in this study that fat-associated aroma can enhance not only fat perception but also salty taste perception (32). Moreover, results obtained from a very large dataset from multiple studies confirmed that salty and fatty sensory attributes were positively correlated, due to perceptual interactions between salty and fatty perception (44). Low-salt cheeses were flavored with aromas associated with salt (sardine), fat (butter), or both (blended). In line with previous studies [e.g., (31)], we found that the sardine salt-associated aroma significantly enhanced salty taste perception in dairy products. Here, we confirmed this odor-induced enhancement effect in cheese products. This result confirmed that the congruence between odor and taste is a determinant factor for the enhancement to occur. Interestingly, we observed that butter aroma also tended to enhance saltiness perception but to a lesser extent than sardine aroma. This suggests that our butter aroma, 2,3-pentanedione, could also carry a salty taste dimension. Several studies have reported that an increase in fat in aromatized model cheeses leads to an increase in saltiness perception (43, 45, 46). Although this effect was explained by physicochemical mechanisms, it cannot be excluded that perceptual interactions between fat and saltiness perception also intervene in the observed saltiness enhancement.

The most surprising result is the absence of odor-induced saltiness enhancement for the blended aroma combining the sardine and butter aroma. Indeed, we expected a significant enhancement of saltiness considering the effect on saltiness of the single aromas. This result can be explained by a change in aroma quality due to the blending and a loss of the salty dimension of the aroma, which is no longer recognized as evoking saltiness by the panelists. We observed that the blended aroma enhanced fat content perception, but surprisingly, the butter aroma had no effect, while the sardine aroma tended to decrease fat perception. A hypothesis could be that a masking effect of fat perception by sardine aroma had occurred.

Finally, we observed that aroma intensity was important in taste enhancement by odor, as suggested in previous studies (30). However, other studies did not find a significant influence of aroma intensity on OISE (29), which suggests that either taste intensity (29) and/or salt or aroma release may play an important role in the enhancement of taste perception by odors. These effects deserve further research.

Very few studies have reported the enhancement of fat perception with aroma. This is the first time that such a cross modal perceptual effect has been clearly demonstrated in cheese matrices. The results reported here differ from those previously reported for model cheeses, where butter aroma was found to significantly enhance fat content perception in most cases (32). However, in this study, it was reported that these effects were highly modulated by the composition and structure of the food matrices. Indeed, two model matrix formulations (low fat, high salt content and high value pH; high fat, low salt content and high value pH) among the eight tested were not found to be able to enhance fat perception despite the addition of a fat-associated aroma (32). These observations are arguments in favor of a high impact of food matrix composition and texture perception on these perceptual effects, as already reported in solid dairy matrices (31).

## Conclusion

Reducing sodium and fat content in cheeses while maintaining sensory quality, consumer acceptability and safety is always a major challenge for the dairy industry. Our study confirmed that until a certain level, it is possible to reduce salt content without impacting the taste or texture profile. We also demonstrated the ability of a salt-associated aroma to compensate for salt reduction in low-salt cheeses. As far as fat perception is concerned, the obtained results were less clear and seemed to be more complex and should be further clarified with additional studies. Thus, this work supports the idea that the addition of congruent aromas alone or in combination with other strategies in dairy products could positively compensate for extra sodium and fat content reduction, as recommended by health organizations. Thus, aroma addition alone or in combination with other strategies can assist the food industry when reformulating low-sodium and low-fat foods while maintaining consumer acceptability. However, although this study was carried out on commercial cheeses, it remains a proof of concept because the results are not directly applicable to cheese production and require prior technological optimizations. Cheese being a fermented product, the first role of sodium chloride in cheese is to regulate and control the fermentations that depend on many variables such as starter strains, inoculation levels, temperatures, cheese moisture and salt level. Moreover, fermentations control ripening process (lipolysis, proteolysis, lactates fermentations, opening etc.) and impact on the different aspect of final quality (texture, functionalities, taste, aroma). Therefore, to generate flavor compounds associated with saltiness and/or fat perception during the maturation of cheeses and to keep them acceptable for the consumers remain a real challenge. Indeed, the nature of the microorganisms constituting the secondary microflora and the technological routes used for the manufacture of cheeses influence their composition, including the production of flavor compounds. Ensuring better control of the organoleptic quality of cheeses implies better knowledge of the different mechanisms involved during their manufacture at technological, biochemical and microbiological levels.

## Data Availability Statement

The raw data supporting the conclusions of this article will be made available by the authors, without undue reservation.

## Ethics Statement

The studies involving human participants were reviewed and approved by the Comité de Protection des Personnes Est-1, N° 2013/64—IDRCB 2013—A01084-41 on 11/21/2013 and Agence Nationale de la Santé et du Médicament, N° B 131283-81 on 11/20/2013. The patients/participants provided their written informed consent to participate in this study.

## Author Contributions

CSa and TT-D contributed to the conception of the study and contributed to the supervision of the study. CSa, TT-D, CSe, and AS contributed to the design of the study, interpreted the data of the work, and contributed to the writing – reviewing and editing of the final draft, and approved the submitted version. CSe and AS contributed to investigation. TT-D and AS performed the statistical analyses. AS wrote the first draft under the supervision of TT-D and CSa. All authors contributed to the article and approved the submitted version.

## Conflict of Interest

The authors declare that the research was conducted in the absence of any commercial or financial relationships that could be construed as a potential conflict of interest.

## Publisher’s Note

All claims expressed in this article are solely those of the authors and do not necessarily represent those of their affiliated organizations, or those of the publisher, the editors and the reviewers. Any product that may be evaluated in this article, or claim that may be made by its manufacturer, is not guaranteed or endorsed by the publisher.
